# Exploring the influence factors and operating mechanisms of age-friendly communities in urban fringe areas from the resilience perspective: a case study in Shangjie Township, Southeast China

**DOI:** 10.3389/fpubh.2025.1624641

**Published:** 2025-08-01

**Authors:** Pinqi Wu, Haiying Liu, Yafeng Zou, Chengfeng Yi, Pingping Du, Yan Song

**Affiliations:** ^1^College of Environment and Safety Engineering, Fuzhou University, Fuzhou, China; ^2^Department of City and Regional Planning, University of North Carolina at Chapel Hill, Chapel Hill, NC, United States

**Keywords:** resilience perspective, age-friendliness, urban fringe communities, structural equation model, operational mechanism

## Abstract

**Introduction:**

The development of age-friendly communities (AFC) is a key initiative in response to the active ageing strategy proposed by World Health Organization. Urban fringe communities (UFC) are characterized by distinctive features such as intricate built environments and heterogeneous resident populations, and these characteristics pose a great challenge to the promotion of age-friendliness in these areas.

**Methods:**

This study combines field surveys and spatial analysis to reveal the age-friendliness in different types of UFC in Shangjie Township, southeastern China. And we employ structural equation model to quantitatively analyze the factors influencing age-friendliness in these communities. Then, this study proposes a mechanism for the operation of AFC in UFA based on the DPSIR model.

**Results:**

Over 50% of the UFC in the study area exhibit satisfactory age-friendliness, while the age-friendliness in some communities still requires urgent improvement. Furthermore, community space, facilities and services, social interactions, and social participation all positively influence the age-friendliness of UFC, with effect sizes ranked in descending order as follows: facilities and services, social interactions, social participation, and community space.

**Discussion:**

This study develops a systematic framework for enhancing the age-friendliness of UFC, providing a viable strategy for addressing the challenges of population ageing. The findings of this study make both theoretical and practical contribution to the development of AFC in UFA.

## 1 Introduction

The current global ageing situation is becoming increasingly severe (1). According to estimates, the proportion of older adults individuals worldwide is expected to reach 12% by 2030 and 16% by 2050 (2). Amid this escalating ageing trend, countries worldwide face the dual challenge of ensuring older adults individuals’ health while maintaining their autonomy and independence (3). In response to the continuous growth of the global older adults population, various countries and regions are dedicated to building safer and more accessible age-friendly communities (AFC) to enhance the quality of life for older adults (4). For instance, American Association of Retired Persons is committed to building livable communities, while UK makes efforts to develop lifetime neighborhoods (5, 6). In 2007, the World Health Organization (WHO) advocated for the development of age-friendly cities in its *Global Age-Friendly Cities Guide*, introducing the definition of AFC for the first time (7). Although many countries adhere to the WHO’s age-friendly city agenda in the development of urban communities, there remains a lack of consensus among institutions and social organizations globally regarding the specific meaning of AFC (8).

In the current research on AFC, under the influence of initiatives proposed by WHO, scholars have explored the concepts from multiple perspectives, including ageing ecology, active ageing, and care for special groups (9–11). Their efforts have contributed to practical advancements in areas such as community infrastructure improvement, promotion of older adults social participation, and support for vulnerable groups (12–14). To promote the development of AFC, scholars have conducted extensive research on evaluating age-friendliness in relation to improving community environments (15), and analyzing factors based on the needs of the older adults (12, 16). These studies identify key factors influencing age-friendliness, including the community environment (17), healthcare services (18), and infrastructure (19). Methods such as multiple regression (20), structural equation model (21), and propensity score matching (82) have been employed to quantify these factors. By exploring how these factors affect the quality of life for the older adults, these studies highlight their potential to enhance both the engagement of older individuals and the overall age-friendliness of the community. In the context of an ageing population and ongoing urbanization, communities are becoming increasingly pivotal in supporting older adults in adapting to these changes.

Urbanization, as another significant global trend, has accelerated the expansion of urban boundaries, gradually blurring the distinction between urban and rural areas. This transformation, coupled with changes in the urban–rural relationship and the growing interpenetration of urban and rural elements, has given rise to the emergence of urban fringe areas (UFA) (22). To accommodate villagers displaced by urban expansion, as well as various mobile populations, urban fringe communities (UFC) have emerged as a unique form of transitional communities from rural to urban (23). These communities grapple with dual pressures of land-use restructuring and ecological degradation (24), while simultaneously experiencing amplified systemic vulnerability due to complex urban–rural interactions, resulting in persistent erosion of community resilience. This underscores the necessity and importance of strengthening internal management within communities to enhance their resilience.

Although extensive research has been conducted on AFC, studies on the age-friendliness of UFA remain limited. In addition, while some scholars have incorporated resilience concepts into research on improving the physical and mental health of the older adults (25), there is a notable paucity of studies applying resilience theory to the development of AFC. As the rapidly ageing population continues to grow, the decline in resilience and physical functioning among older adults has led to more diverse needs regarding the age-friendliness of their communities. In addition, UFC, owing to their distinct spatial and geographical characteristics, experience high population mobility and possess a diverse resident profile. The rapid expansion of land for community development further accelerates the rate of change in these communities. These combined factors make it difficult for older adults to adapt to their communities and maintain stable social ties. As a result, their sense of community attachment and mental wellbeing declines, highlighting the urgent need to improve community age-friendliness. However, the unique characteristics of UFC, combined with their insufficient resilience, pose obstacles to achieving AFC. Therefore, in response to the challenges of the ageing society, it is essential to strengthen community resilience to meet the diverse needs of older adults and enhance the age-friendliness of communities.

The concept of resilience originated in the field of physics. Holling introduced it to ecosystems in 1973, and defined it as the ability to maintain the stability of ecosystems (26). As resilience has been increasingly applied in urban studies (27), economics (28), and gerontology (29), community resilience gradually emerged as a critical issue, as communities are the basic units of urban systems (30). Against this backdrop, the vulnerability of UFC and its multidimensional impact on the health of the older adults residents urgently require attention. UFC should enhance their older adults-friendliness to improve the quality of life of older adults residents and support the integration and adaptation of older adults immigrant groups.

China is undergoing rapid population aging, with individuals aged 65 and above accounting for 13.5% of the total population in 2020 according to the “*Seventh National Population Census Bulletin*.” As a representative coastal city in southeastern China, Fuzhou serves as the political, economic, and cultural hub of Fujian Province while also functioning as a major destination for population migration. According to the *2020 Fuzhou Population Census Yearbook*, the proportion of older adults individuals over 65 in Fuzhou is 11.72%, indicating that the city has officially transitioned into an aging society despite its slightly lower aging rate compared to the national average. Compared to most western countries with higher aging rates, Fuzhou’s aging process demonstrates distinctive characteristics such as “ageing before becoming wealthy.” Its aging process differs both from the gradual aging pattern of western countries built upon highly developed economic foundations and from the “growing old while growing rich” development path seen in China’s megacities. Furthermore, Fuzhou’s typical marginal areas possess relatively complete peripheral characteristics, which consequently manifest especially age-friendly challenges. Of particular note is that, under the dual influence of spatial spillover from the central urban area and innovation-driven development in the high-tech industrial zone, Shangjie Township has gradually developed into a typical UFA of Fuzhou, with its communities serving as important case studies for research on the construction of age-friendly UFC.

The structure of this paper is as follows. The second section defines the concept of AFC in UFA from a resilience perspective. The third section introduces the data sources and outlines the main research methods. The fourth section presents the empirical analysis, including the current status of age-friendliness in UFC and the key influencing factors. Finally, the final section provides the main conclusions and policy recommendations.

## 2 Age-friendly communities in urban fringe areas from a resilience perspective

The concept of AFC can be traced back to the “elder-friendly communities” proposed by the Visiting Nurse Service in New York. It emphasizes that communities should enable older residents to actively participate in community activities, maintain their independence, and reduce the risk of isolation (31). As the older adults population continues to increase, concepts such as “livable communities” (5) and “lifetime neighbourhoods” (6) have emerged in response to the need of creating environments that are conducive to older adults living. These concepts aim to extend the years of independent living for older adults and promote ageing in place. Population ageing brings about many fundamental issues such as health care and social security. To comprehensively promote the participation of older people and further improve their wellbeing, WHO proposed the concept of active ageing (32), which is “the process of making greater efforts to improve the quality of life in old age in the areas of personal health services, social participation, and public safety.” In 2007, WHO defined AFC from the perspective of active ageing, and proposed eight core elements in the *Global Age-Friendly Cities Guide* (7). It emphasized that these communities should provide health and safety services and opportunities for social participation, encouraging active ageing to enhance the quality of life for older adults. The definition and basic elements of AFC proposed by WHO incorporate the idea of active ageing, which has laid a better policy foundation and implementation framework for subsequent research by scholars.

Building on earlier frameworks, researchers have further defined AFC. They argue that an AFC should: provide infrastructure and service support that meets the needs of older adults (33); create a barrier-free and inclusive social environment that enables the active social participation of older adults to enhance their health and wellbeing (34); cater for the different needs of special groups of older adults, providing conditions and platforms to help them actively integrate into other social groups (35). Special groups of older persons mainly include marginalized and vulnerable older adults, such as older women, empty nesters, and migrant older adults. While considerable advancements have been made in the study of AFC, the majority of research remains confined to the traditional urban–rural dichotomy analytical framework, overlooking the distinctive challenges of the urban–rural transition zone. As a special transitional form in the process of urbanization, UFC exhibit dual urban–rural attributes. They are characterized by differentiated living spaces, challenges in community governance, diversified economic structure, cultural conflicts between urban and rural areas, and a complex resident composition (36, 37). Although China’s UFC share similarities with urban sprawl in Western cities, they have developed unique local characteristics in the context of rapid urbanization (38), such as innovative governance models like villages into residential communities. Furthermore, to achieve sustainable development in UFA, China advocates implementing a management system based on “adapting to local conditions,” “establishing a multi-stakeholder governance mechanism led by the government,” and “implementing coordinated governance.”

Due to the impacts of spatial dislocation (39), the unique characteristics and complexities of the living environment result in increased diversity in the social characteristics of older adults within UFC, heightening their sensitivity and vulnerability. Specifically, the decline in physical function and the accompanying reduction in adaptability among older adults lead to a chain reaction that manifests their vulnerability as individuals (40). Additionally, due to the spatial and social characteristics of UFC, older adults residents face amplified risks of cultural identity challenges and social isolation, further increasing their vulnerability (41). Therefore, enhancing community resilience to assist in the physical and mental adaptation of the older adults has become imperative. Community resilience refers to the ability of a community system to maintain its core functions through the synergistic effects of spatial adaptability, social self-organization, and sustainable development when responding to pressures such as natural disasters and social change (42). It is a combination of stability, adaptability, and recovery capacity (43). In the context of community resilience, adaptability refers to the ability of a community to withstand external disasters (44); adaptability refers to the ability of a community to change its structure after major events or changes and to some extent anticipate similar events (45); recovery refers to the ability of a community to return to normal operations after experiencing external shocks (46). AFC align with these aspects by emphasizing community stability, adaptability, and recovery capacity, specifically manifested as follows: First, a stable community environment can provide a safe residence for the older adults, reducing their anxiety and insecurity, while allowing them to maintain existing social networks and promoting social interactions among seniors. Second, the importance of community adaptability is reflected in its ability to help older adults adjust to new environments and adopt new lifestyles, better enabling them to cope with changes brought about by physical decline. Third, community recovery capacity is particularly crucial in responding to external shocks such as natural disasters, public health events, and human-induced damage, and the rapid restoration of community facilities and services can meet the basic living needs of older adults. Meanwhile, the community’s ability to repair seniors’ social networks is equally important.

**Figure 1:**
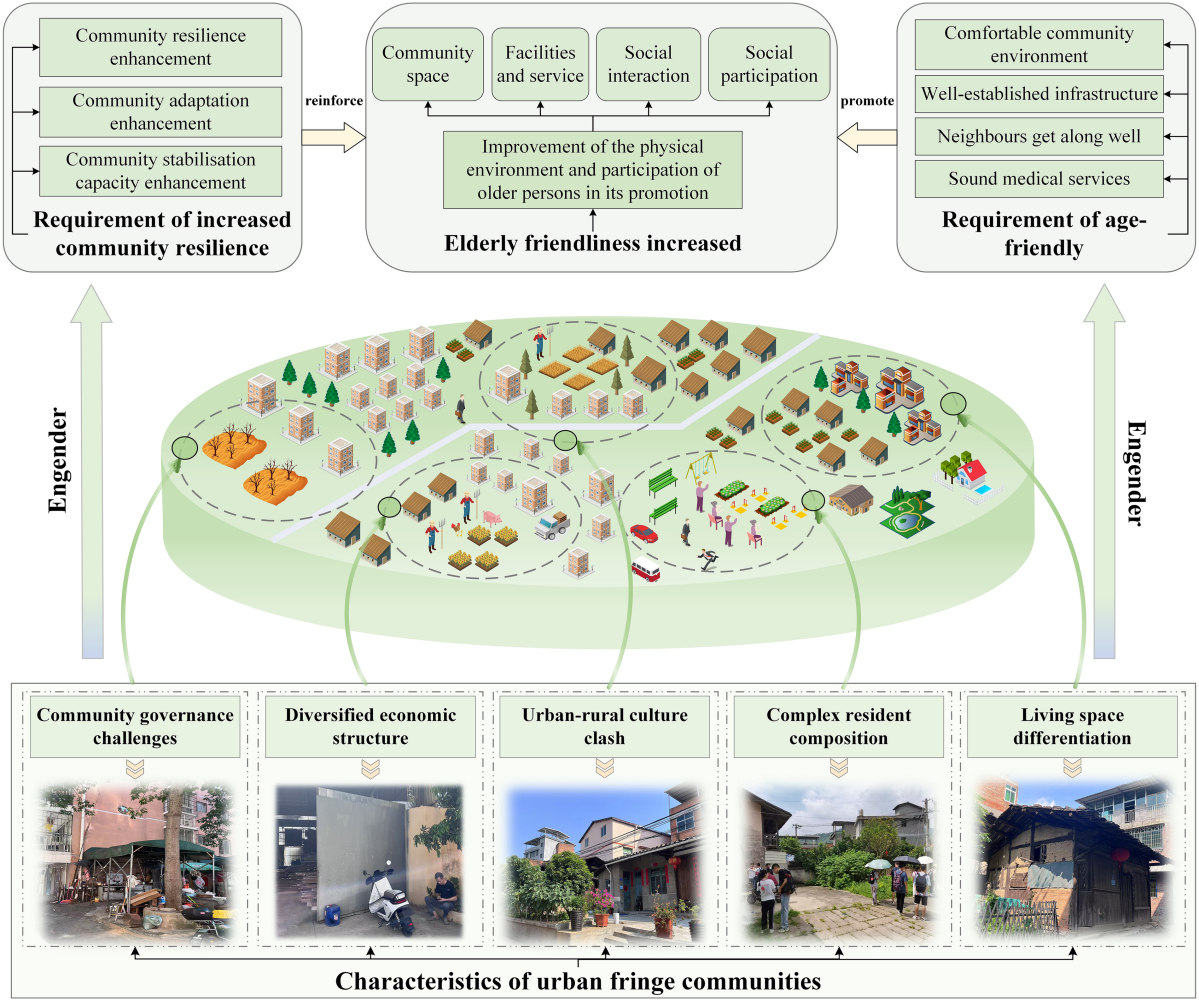
Schematic of AFC in UFA from a resilience perspective.

In this regard, enhancing community stability, adaptability, and recovery capacity has become a shared goal for strengthening community resilience and achieving AFC. Exploring the creation of AFC in UFA from a resilience perspective ensures both scientific rigor and practicality. The vulnerability exhibited by older adults necessitates that, in addition to improving their own adaptability, communities should also make corresponding efforts to address the barriers that hinder their ability to cope with physical functional decline (47). The consideration of community resilience aimed at being age-friendly should focus on the community’s adaptability in responding to active ageing and its ability to maintain sustainable development. Improving age-friendliness in UFC, considering their current state, should focus on enhancing the physical environment of the community and increasing its capacity to support older adults’ participation. The physical environment primarily includes the living spaces, basic infrastructure, and services of the community, while older adults’ participation manifests through their social interactions and involvement. To this end, AFC in UFA based on a resilience perspective should be able to build a resilient community environment with adaptability, resilience and stability through spatial restructuring, optimization of facilities and services, promotion of interaction, encouragement of participation and cultural integration. Specifically, at the community space level, efforts should be made to create comfortable community living spaces and outdoor spaces; at the facilities and services level, it is essential to strengthen infrastructure construction and improve community public service systems; at the social interaction level, initiatives should focus on promoting interaction among older adults residents and integrating smart elements to help them access information and seek advice; at the social participation level, opportunities should be provided for senior residents to engage in employment and volunteer activities, with parallel efforts to promote cultural integration. This community development mode not only addresses the challenges posed by the physical decline of the older adults, but also addresses the identity crisis caused by community transformation. By establishing a resilient support system, it helps the older adults cope with the dual challenges of sudden events and community transformation (Figure 1).

## 3 Materials and methods: study area and research design

### 3.1 Research ideas

**Figure 2:**
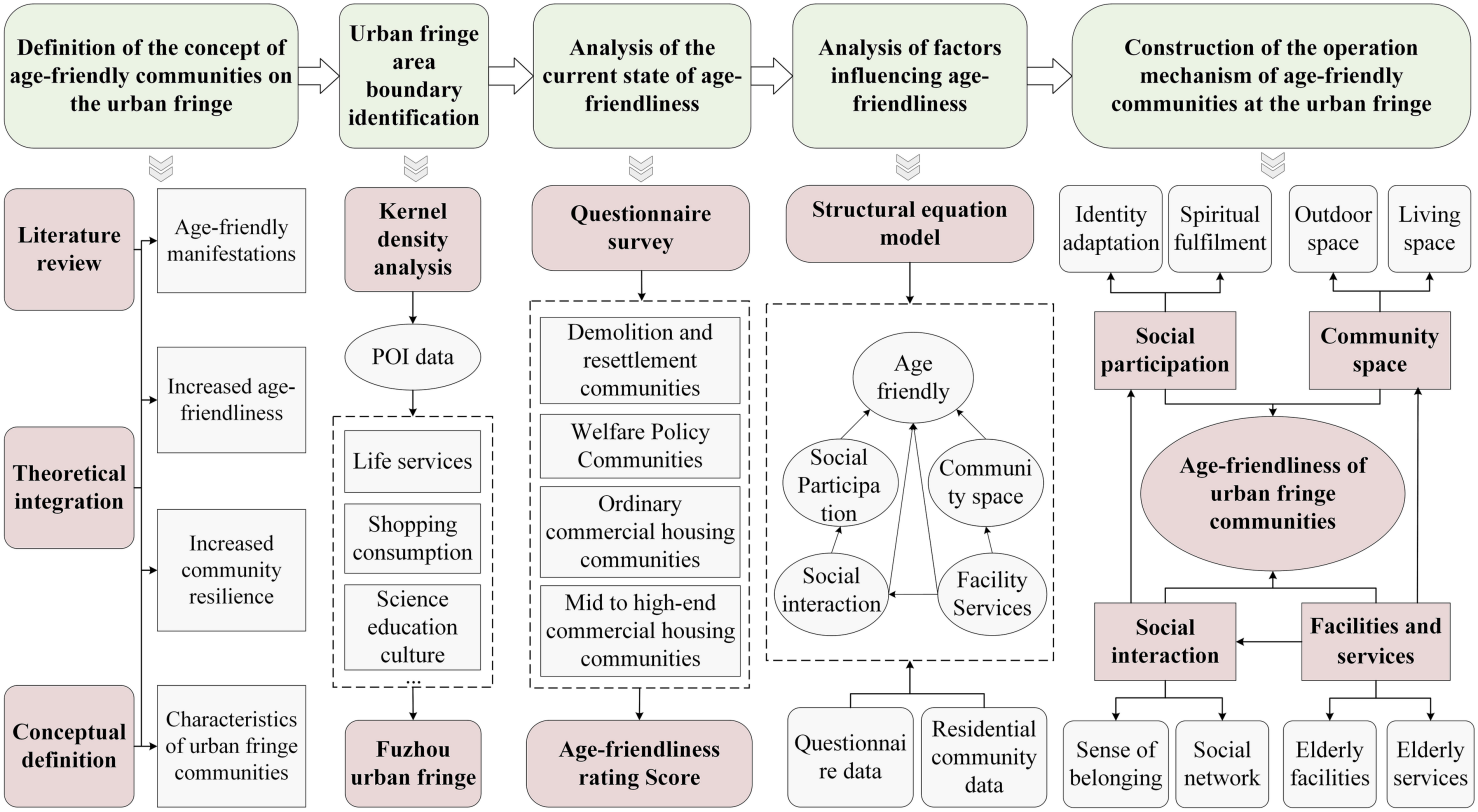
The research idea diagram.

Against the backdrop of accelerating urbanization and a growing older adults population, improving the physical and mental health of older adults is of paramount importance. However, UFC face challenges in creating age-friendly environments due to their lack of resilience. To systematically enhance the age-friendliness of UFC, this study first defines the conceptual framework of AFC in UFA from a resilience perspective. Subsequently, leveraging POI data, the boundaries of Fuzhou’s UFA are identified using kernel density analysis and the Densi-Graph method. Within these delineated areas, residential communities served as the fundamental units of analysis. Thereafter, based on field survey questionnaire data and online rating metrics from these communities, the current state of age-friendliness in urban fringe communities is evaluated through the natural breakpoint method. Subsequently, a structural equation model (SEM) was constructed to examine the specific influencing effects and pathways through which community space, facilities and services, social interaction, and social participation affect age-friendliness in UFC. Finally, we qualitatively elucidate the influencing mechanism responsible for AFC in UFA by employing the DPSIR model (Figure 2).

### 3.2 Study area

Shangjie Township is located in the central part of Fujian Province, southeastern China, and the administrative area covers 139.16 km^2^. According to the “*Fuzhou Urban Land Spatial Master Plan (2021–2035)*” (hereinafter referred to as “*the Plan*”), Shangjie Township is located on the edge of the main urban core area and is part of the urban fringe of Fuzhou. Aside from the High-tech Zone, Shangjie Township contains 18 villages. According to the “*Shangjie Township Seventh National Population Census Bulletin*,” Shangjie Township has a permanent population of 197,399, of which 32,732 are aged 60 or above, making up 16.58% of the total population. This highlights a relatively severe ageing problem. While the public transport system basically covers the entire town, certain old villages still have narrow roads and rely on rural passenger transport. The construction of Fuzhou University Town began in 2001, and Shangjie Township is situated within the boundaries. As the university town has developed, Shangjie Township has gradually emerged as a typical example of Fuzhou City’s UFA. At the same time, with the economic development of the high-tech zone and the gradual development of the university town, Shangjie Township has gradually formed an economic development mode driven by higher education institutions and the integration of industry and urban development. This has led to a large number of demolition and construction projects, with many villagers losing their original residences and land and moving into resettlement communities. However, a significant number of residents have retained their original housing and remained in rural communities. As a result, this case study area exhibits the typical characteristics of an urban–rural fringe area.

**Figure 3:**
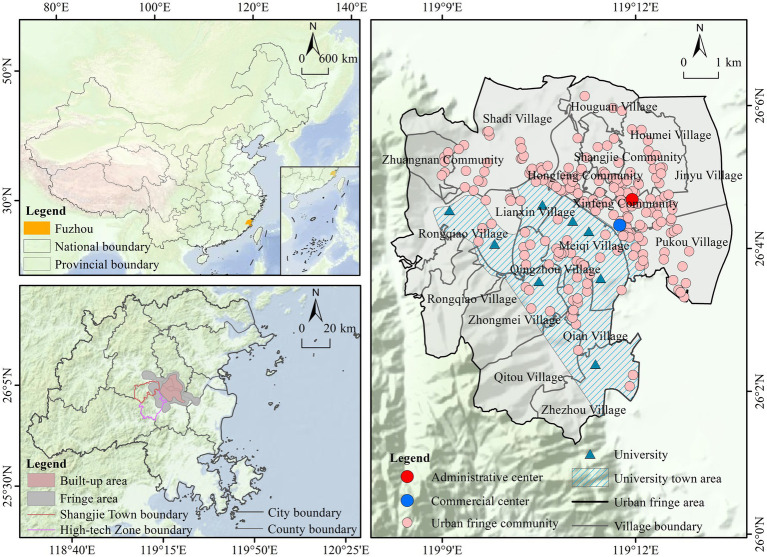
Location of the study area.

This study builds on “*the Plan*” framework to identify the UFA of Fuzhou City using point of interest (POI) data through kernel density analysis combined with the Densi-Graph method. Figure 3 shows that, except for Xiyuangong Village, all administrative villages in Shangjie Township are located within the UFA. And the urban fringe demarcation shown in the right panel of Figure 3 represents the cartographic output for Shangjie Township with Xiyuangong Village removed from the analysis. Xiyuangong Village, with its mountainous terrain and preserved rural landscape, is not classified as an UFA. The boundary definition of UFC needs to take both social and spatial attributes into account. This study uses spatial attributes as the primary basis while considering social attributes, and treats the communities located in the UFA of Shangjie Township as UFC. Among them, UFA refers to geographical areas with urban–rural transition characteristics, and UFC specifically refers to basic residential units within the scope of UFA. Additionally, since neighborhoods are a specific form of community, this study takes the various neighborhoods within the UFA of Shangjie Township as the basic data units, analyzing their current status and influencing factors in terms of age-friendliness. Based on this, the study also explores the construction of an operational mechanism for AFC.

### 3.3 Data sources

The vector data used in this study were obtained from the Fuzhou Minhou County Natural Resources and Planning Bureau, and the Shangjie Township government provided the socioeconomic and cultural data related to Shangjie Township. We collected residential community data through Python programming, obtaining 202 community points with geographic coordinates in WGS1984 from the Anjuke website. Furthermore, we conducted field research and distributed community questionnaires to capture older adults’ lived experiences and residential community profiles. In July 2024, a total of 400 questionnaires were distributed in various UFC of Shangjie Township, with 332 valid responses collected. The questionnaire mainly covers five aspects: (1) basic information of respondents, (2) satisfaction with community living space, (3) satisfaction with community facilities and services, (4) feelings about social interaction, and (5) frequency of social participation. The participants are mainly non-working older adults people aged between 60 and 90, residing in various housing types such as commercial housing, rental housing, public housing, etc. Their living arrangements vary, including living alone, with children or living with spouses.

The POI were collected from the Amap Open Platform.^1^ It includes 10 categories of POI data closely related to urban and socioeconomic activities, such as life services, education and culture, shopping, and consumption. This study employs POI data to delineate the boundary of the UFA in Fuzhou. UFC, characterized by a high flow of population, intersecting community management systems, underdeveloped infrastructure, significant land development intensity, and diverse community cultures, represent a multidimensional and unique social space. There is currently no unified standard for defining their boundaries. To avoid arbitrary selection of communities, we chose neighborhoods within the identified UFA as the subjects in this study.

### 3.4 Methods

#### 3.4.1 Densi-Graph method

Densi-Graph is a method for analyzing density variations (48). This study employs the Densi-Graph method to delineate UFA in Fuzhou City. This method determines the scope of urban fringe areas by analyzing the relationship curve between kernel density values and the theoretical radius of corresponding closed curves. To be specific, in the relationship curve between density values and theoretical radius, the curve within urban areas is a horizontal straight line, while the curve shows a steep upward trend when transitioning from urban to rural areas. Consequently, the inner boundary of the urban fringe area was delineated by the density isopleth corresponding to the first fluctuation point after stabilization of the radius increment curve, while the outer boundary was defined by the density isopleth at which the growth trend became irreversible. The area enclosed between these two boundaries was ultimately identified as Fuzhou’s urban fringe zone.

#### 3.4.2 DPSIR model

The DPSIR model consists of five components: driving force, pressure, state, impact, and response. And it is the combination and optimization of Pressure-State-Response (PSR) model and Driver-State-Response (DSR) model. The DPSIR model can systematically show the causal chain of problems and help to analyze the interrelationships between different factors (49, 50). Therefore, this study employs DPSIR model to analyze the operation mechanism of age-friendly communities in urban fringe areas. Specifically, by analyzing the driving forces behind age-friendly changes in UFC, we can identify the pressures facing age-friendly improvements and the state of the community under these pressures, determine the impact of this state on the community, and propose specific response measures. The implementation of specific response measures should be combined with the main impact dimensions identified by the SEM to propose targeted measures, providing a quantitative analysis basis for the DPSIR model.

#### 3.4.3 Structural equation model

##### 3.4.3.1 Model setting

Structural equation model is a multivariate data analysis method that can simultaneously consider the relationships between multiple variables (51). This study employs structural equation model to analyze the factors influencing age-friendliness in UFC. Age-friendliness is closely related to safety, comfort, and emotional belonging within the community. Grounded in the concept of resilience, this study proposes that AFC in UFA enhance age-friendliness through four key dimensions: community space, facilities and services, social interactions, and social participation. Besides, existing research indicates that community age-friendliness aims to enhance the quality of life for older adults and facilitate their social interaction and participation, which in turn improves mental health (44). Furthermore, the quality of life for older adults is closely linked to well-developed community infrastructure and public services, with adequate housing space and comfortable outdoor areas also playing a significant role (52). Thus, this study proposes the following hypotheses: Community space has a direct positive impact on age-friendliness (D1); Facilities and services have a direct positive impact on age-friendliness (D2); Social interaction has a direct positive impact on age-friendliness (D3); Social participation has a direct positive impact on age-friendliness (D4).

**Figure 4:**
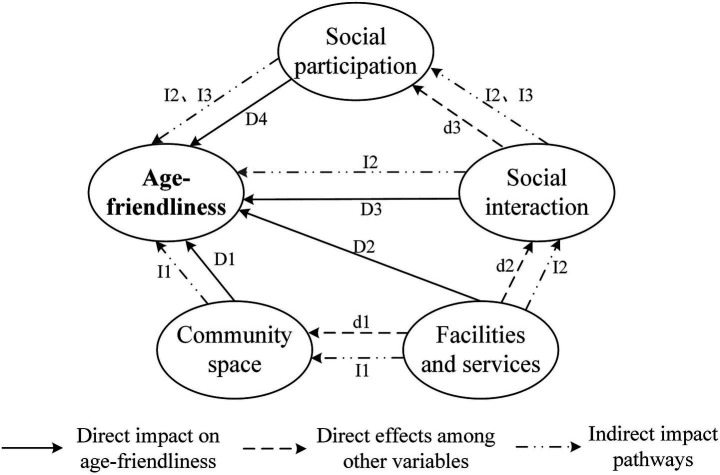
Modeling of age-friendly impact pathways in UFC.

Furthermore, this study further analyzes the interactions among the four dimensions. In UFC, small parks and outdoor fitness areas serve as primary leisure spaces for older adults, which can, to some extent, promote their daily social interactions (53). Additionally, older adults can access information about employment and volunteer opportunities through close daily social interactions, providing them with possibilities for social participation. UFC are characterized by the mutual influence of urban and rural areas, exhibiting dynamic spatial interactions between these zones. While areas with higher community density tend to have more comprehensive facilities and services, peripheral suburban regions face spatial deficiencies. To this end, this study proposes the following hypotheses: d1: The provision of facilities and services will promote social interactions among older adults; d2: Close social interactions will encourage older adults to engage in social participation; d3: Facilities and services influence the allocation of community space. Based on existing literature, this study proposes a model hypothesis from two levels: community background conditions and the needs of the older adults themselves. The impact pathways include the direct effects of four latent variables—community space, facilities and services, social interaction, and social participation—on age-friendliness (D1, D2, D3, D4), the direct effects between the latent variables (d1, d2, d3), and their indirect effects on age-friendliness (I1, I2, I3) (Figure 4).

##### 3.4.3.2 Variable selection

This study adopts a resilience perspective and uses SEM to quantitatively analyze the factors influencing the age-friendliness of UFC. Community space, facilities and services, social interaction, social participation, and age-friendliness are set as latent variables in the model. The enhancement of community resilience and the improvement of age-friendliness are primarily achieved by increasing the community’s stability, adaptability, and recovery capacity. Therefore, the observed variables included in each latent variable correspond to measuring the community’s stability, adaptability, and recovery capacity during variable selection. Community age-friendliness from a resilience perspective should ensure the safety and comfort of the community as well as the emotional support of the older adults, and to this end, community safety, community comfort, and emotional belonging are used as age-friendliness observational variables to measure community stability, adaptability, and recovery capacity, respectively. At the community space level, it is emphasized that the community should provide a spatially satisfying and comfortable living environment for the older adults (54), and that the occupancy of community space also affects the space for the older adults in their daily lives. Therefore, satisfaction with living space, comfort of living space, and recovery of space occupancy are used as observational variables to measure the stability, adaptability, and recovery capacity of community space, respectively. At the level of facilities and services, communities should meet the basic needs of older adults by providing a complete range of infrastructure types (55) and ensuring that daily transportation needs are satisfied (56). At the same time, with the development of home-based older adults care services, “property-based older adults care” has gradually emerged (57). The sustainability of property services is beneficial for promoting the physical and mental wellbeing of older adults individuals living alone. Therefore, completeness of facility types, convenience of transportation, and sustainability of property services are used as observation variables to measure the stability, adaptability, and recovery capacity of facilities and services. At the level of social interactions, frequent influx and outflux of residents can hinder the establishment of stable social networks for older adults. Close neighborhood interactions play an important role in promoting the physical and mental wellbeing of the older adults (58). In the smart era, convenient access to information and consultations can help older adults maintain or restore their existing social relationships, making it an important measure of age-friendliness. Therefore, turnover rate of new and old residents, closeness of neighborhood interactions, and convenience of information access are used as observation variables to measure the stability, adaptability, and recovery capacity of social interactions. At the level of social participation, the reemployment of older adults individuals (59) and opportunities for older adults to engage in community affairs can meet their need for self-actualization (60). Additionally, in UFC, there is a higher presence of outsiders, leading to significant cultural differences. The transmission of traditional culture can also promote social participation among the older adults to some extent (61). Therefore, availability of job opportunities, satisfaction with the cultural environment, and degree of participation in community affairs are used as observational variables to measure the stability, adaptability, and recovery capacity of older adults’ social participation. Table 1 details the model variables.

**Table 1:** Selection of variables.

Latent variable	Observed variable	Variable calculation
Age-friendliness	Community safety	Questionnaire scoring
	Community comfort	Questionnaire scoring
	Emotional belonging	Questionnaire scoring
Community space	Satisfaction with living space	Questionnaire scoring
	Comfort of living space	According to the Anjuke rating scale
	Recovery of space occupancy	Questionnaire scoring
Facilities and services	Completeness of facility types	Graded according to the number of POIs in the 300-m buffer zone
	Convenience of transportation	Graded according to distance from metro stations and bus stops
	Sustainability of property services	Questionnaire scoring
Social interaction	Turnover rate of new and old residents	Questionnaire scoring
	Closeness of neighborhood interactions	Questionnaire scoring
	Convenience of information access	Questionnaire scoring
Social participation	Availability of job opportunities	Combine the questionnaire and number of companies in the 300-m buffer zone
	Satisfaction with the cultural environment	Classification according to number of cultural sites and activities
	Degree of participation in community affairs	Questionnaire scoring

## 4 Results

### 4.1 Analysis of the current situation of age-friendliness in UFC

**Figure 5:**
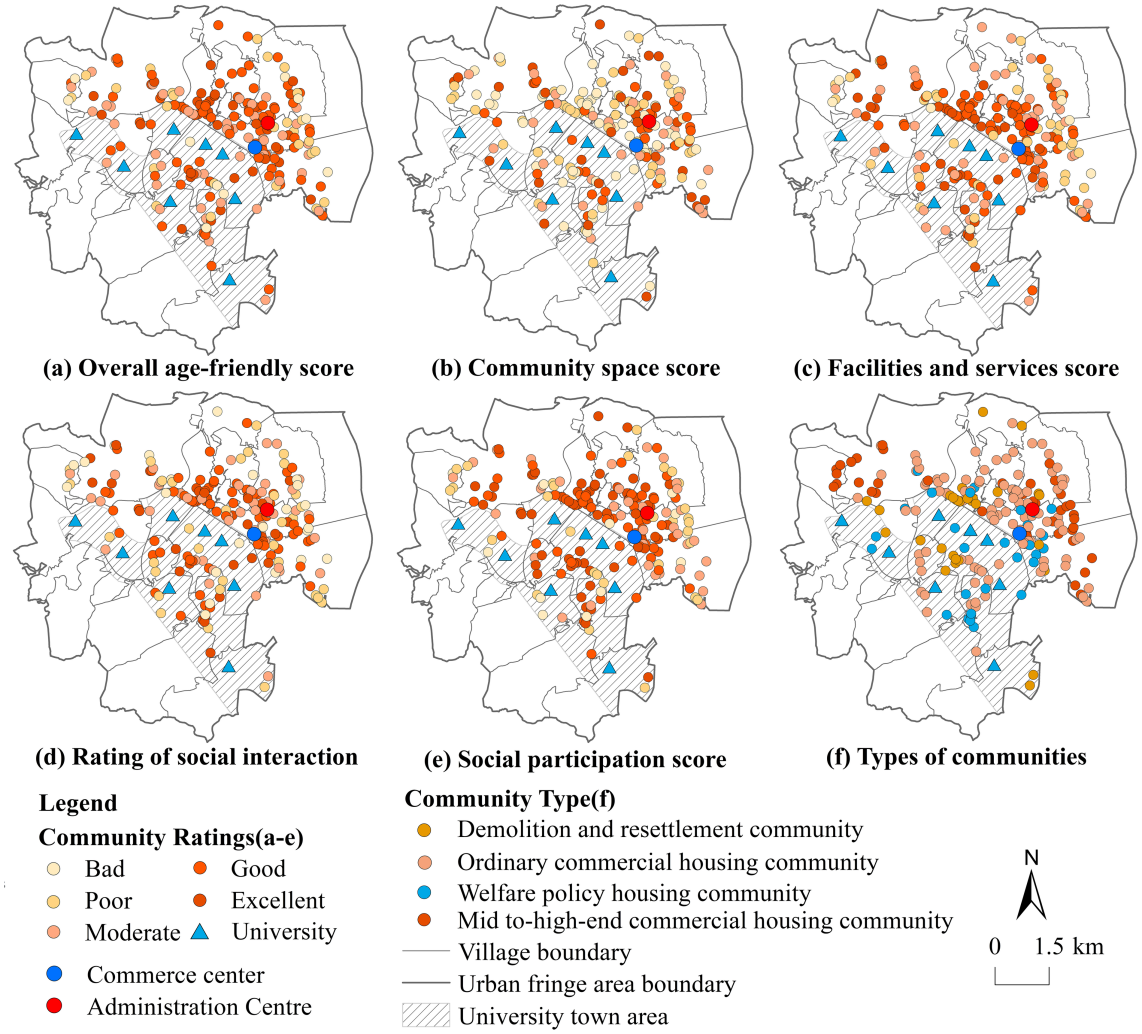
Results and spatial distribution of dimension scores for different types of UFC.

There is currently no unified community classification standard. Based on existing literature on community typology, combined with the current socio-economic development status of Shangjie Township and the results of field research on communities, the 202 community points in Shangjie Township can be categorized into four types: demolition and resettlement communities, welfare policy housing communities, ordinary commercial housing communities, and mid to high-end commercial housing communities (62). Their respective numbers are 29, 31, 103, and 39, accounting for 14.36, 15.34, 50.99, and 19.31% of the total number of the communities. By combining data from community field research questionnaires and the scoring results obtained for the residential community data, and using the natural breakpoint classification method in ArcGIS, the overall scores for older adults-friendly environments, community space, facilities and services, social interactions, and social participation in UFC of Shangjie Township are categorized into five levels: excellent, good, moderate, poor and bad (Figure 5).

Based on the score ranges for each level in the overall scores for older adults-friendly environments, we analyzed the older adults-friendly ratings for four different types of communities. The analysis results show that the quality ratings of the four types of communities are as follows. First, in demolition and resettlement communities, the number of communities at each level—excellent, good, moderate, poor, and very poor—are 6, 11, 7, 4, and 1, respectively. Second, in welfare policy housing communities, the numbers are 8, 9, 10, 2, and 2. Third, in ordinary commercial housing communities, the numbers are 19, 39, 28, 11, and 6. Finally, in mid-to-high-end commercial housing communities, the numbers are 5, 15, 9, 5, and 5. The proportion of communities with a good or higher rating for age-friendliness among the four types is 58.62, 54.84, 56.31, and 51.28%, respectively.

The results above indicate that resettlement communities have relatively high age-friendliness. Field survey and analysis results (see Figure 5) show that, the demolition and resettlement communities where older adults residents live offer more convenient facilities compared to their original rural living environments. Additionally, the resettlement process is conducted on a village basis, allowing older adults individuals to remain familiar with one another, which increases their opportunities for social interaction. Welfare policy housing communities and ordinary commercial housing communities share common characteristics, including relatively complete facilities, a moderate distance to public service facilities, and convenient access to those facilities. At the same time, most communities are relatively densely distributed, providing more opportunities for older adults social interactions and participation, resulting in a correspondingly higher level of age-friendliness. The age-friendliness of mid to high-end commercial housing communities is relatively low. Among these communities, those located in densely populated areas and close to administrative, commercial centers, and clusters of enterprises tend to have better age-friendliness compared to those situated in more remote locations and farther from university towns.

### 4.2 Analysis of factors influencing age-friendliness in UFC

**Figure 6:**
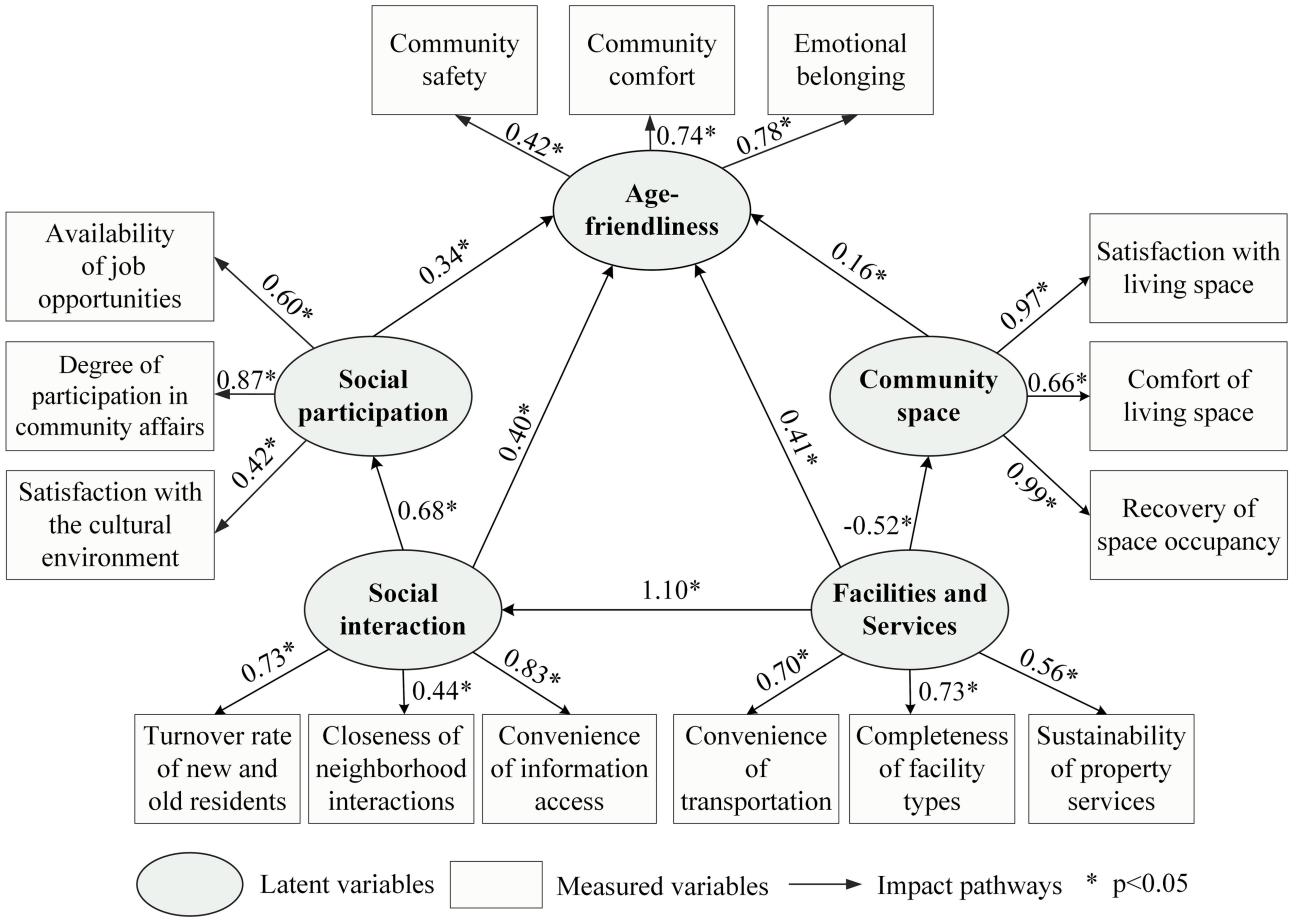
Mechanisms of the influence of different factors on community age-friendliness.

Figure 6 presents the impact paths, path coefficients, and significance of community space, facilities and services, social interaction, and social participation on age-friendliness. It is evident that community space and social participation have a direct impact on age-friendliness, facilities and services, as well as social interaction, exert both direct and indirect effects on age-friendliness.

To further analyze the specific effects of each latent variable on age-friendliness, this study calculated the direct effects, indirect effects, and overall effects of each latent variable on age-friendliness, as shown in Table 2. The calculation of indirect effects is specifically manifested as follows: An increase of one standard deviation in the level of facilities and services is associated with an increase of 1.10 standard deviations in social interaction. Additionally, an increase of one standard deviation in social interaction is associated with an increase of 0.40 standard deviations in age-friendliness. Therefore, in the pathway “facilities and services → social interaction → age-friendliness,” the indirect effect of facilities and services on age-friendliness through social interaction is 0.44 (1.10 * 0.40). The same applies to other indirect effect values. Overall, community space, facilities and services, social interaction, and social participation all have a positive correlation with age-friendliness. Among these, the impact of community facilities and services on age-friendliness is the greatest (1.02), followed by social interaction (0.63), then social participation (0.34), and finally community space (0.16).

**Table 2:** Results and effects of latent variables.

Latent variable	Impact pathways	Types of effect		
		Direct effect	Indirect effect	Total effect
Community space	Community space → age-friendliness	0.16		0.16
Facilities and services	Facilities and services → age-friendliness	0.41		1.02
	Facilities and services → community space	−0.52		
	Facilities and services → social interaction	1.10		
	Facilities and services → community space → age-Friendliness		−0.08	
	Facilities and services → social interaction → age-friendliness		0.44	
	Facilities and services → social interaction → social participation → age-friendliness		0.25	
Social interaction	Social interaction → age-friendliness	0.40		0.63
	Social interaction → social participation	0.68		
	Social interaction → social participation → age-friendliness		0.23	
Social participation	Social participation → age-friendliness	0.34		0.34

### 4.3 Operation mechanism of age-friendly communities in urban fringe areas based on DPSIR model

**Figure 7:**
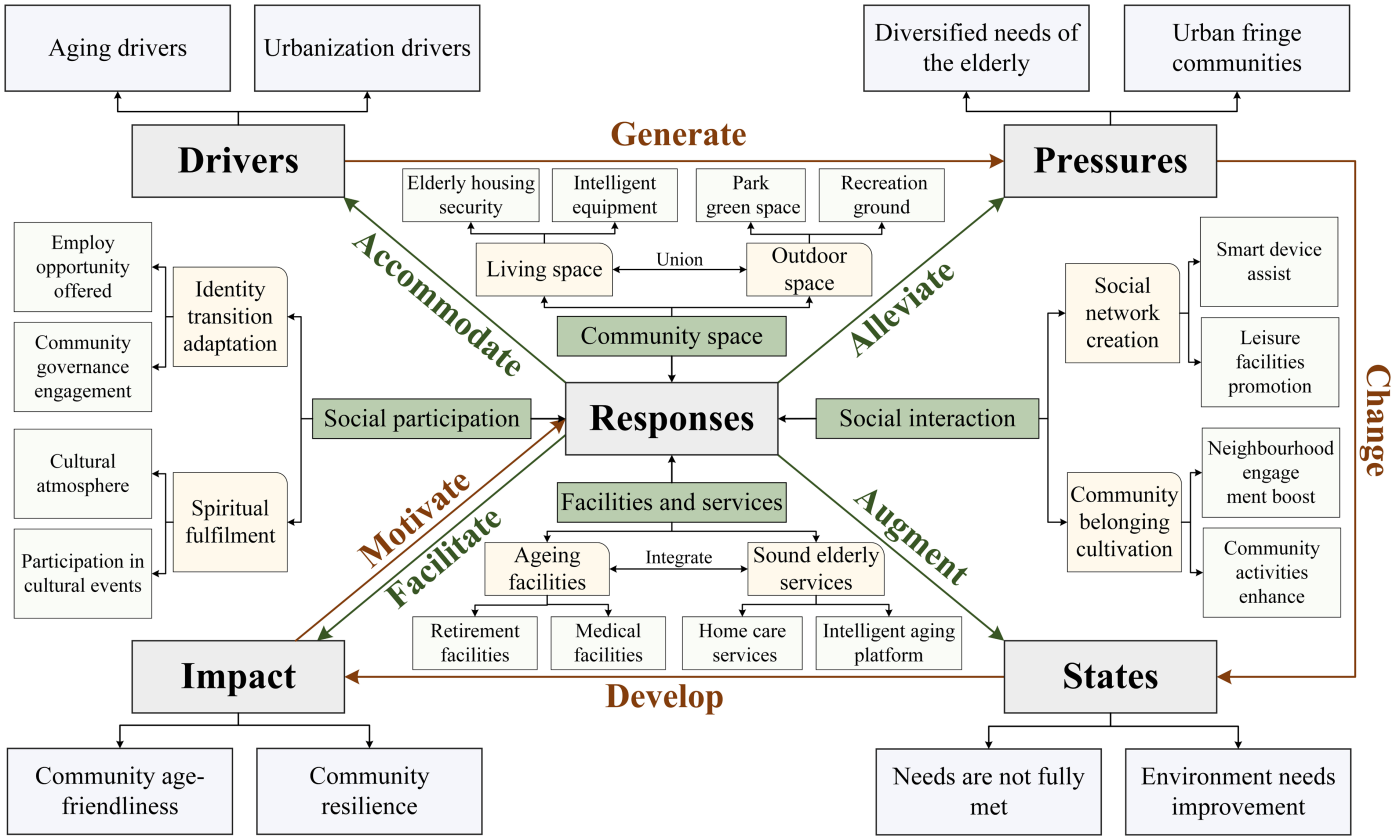
Schematic diagram of the operational mechanism of AFC in UFA.

This study proposes an operational framework for AFC in UFA based on the DPSIR model and field investigation. Under the dual pressures of ageing and urbanization, there is an increasing diversity in older adults needs and rapid growth of UFC. These pressures result in unmet needs for the older adults and highlight the urgent need for environmental improvements, which in turn affect both the age-friendliness and resilience of the community. To address these impacts, targeted measures should be implemented to regulate driving factors, alleviate pressures, improve existing conditions, and mitigate negative effects, thereby achieving age-friendly development in UFC (Figure 7).

Specifically, population ageing and urbanization are the main drivers, with a growing older adults population and expanding residential areas creating a mismatch between the resources available in UFC and the growing diversity of older people’s needs. However, the current situation of unmet older adults needs and the complex socio-spatial environment in UFC collectively exert negative impacts on the enhancement of both age-friendliness and community resilience. To steer UFC toward age-friendly development, responsive measures should focus on four key dimensions: community space optimization, facility and service enhancement, social interaction facilitation, and social participation promotion. The results of the SEM indicate that these four dimensions have the strongest explanatory effects on the observed variables of recovery of space occupancy, completeness of facility types, convenience of information access, and degree of participation in community affairs, respectively. Therefore, improving these aspects should be prioritized to enhance the age-friendliness of communities. Accordingly, at the community space level, efforts should primarily focus on improving the recovery capacity of spatial occupancy, strengthening age-friendly spatial modifications, and installing barrier-free facility systems to ensure the safety of older adults. At the facility and service level, priority should be given to improving community infrastructure and optimizing the public service system to mitigate the negative effects of age-related physical decline. In terms of social interaction, it is essential to strengthen volunteer-led training programs on smart device usage. The integration of smart technology can both expand older adults’ access to information and help them maintain their existing social networks. Regarding social participation, emphasis should be placed on establishing mutual aid organizations for older adults to encourage their involvement in community affairs. At the same time, employment opportunities should be provided to the older adults based on their needs to achieve multi-dimensional development from basic participation to value creation.

## 5 Discussion

This study examines community age-friendliness in typical UFA while incorporating community resilience into the AFC framework, thereby expanding the research on age-friendly development in UFA. This dual research perspective enhances theoretical understanding of age-friendliness at the community level. This study analyses the current state of age-friendliness in UFC with the aim of assessing the status of community-based age-friendly environment construction and promoting external forces to jointly enhance the age-friendliness of communities. Furthermore, this study conducts a quantitative analysis of various factors affecting community age-friendliness, determines the degree of influence of these factors, and proposes operation mechanisms for enhancing age-friendliness in UFC, aims to provide valuable references for improving the age-friendliness of such communities.

### 5.1 Pathways to improving age-friendliness in different types of urban fringe communities

Based on field-based questionnaires conducted across UFC within the research area, this study identifies significant heterogeneity in older adults-friendliness among four distinct community typologies. Different types of UFC require tailored approaches to improve age-friendliness for demolition and resettlement housing communities, the internal infrastructure is relatively inadequate compared to their counterparts in urban core areas, and related supporting facilities are also lagging behind (63). Therefore, priority should be given to strengthening community infrastructure construction, with urgent needs such as elevator installation and barrier-free pathway renovations addressed first. For ordinary commercial housing communities experiencing high population mobility which hinders older adults residents’ ability to establish stable social connections, the focus should shift toward facilitating social interactions to enhance adaptation (64). Welfare policy housing communities in UFA benefit from government-subsidized low rents and maintenance fees. However, they exhibit significant gaps in accessibility infrastructure and green space coverage compared to their urban-core counterparts (65). Consequently, targeted interventions should prioritize upgrading physical facilities and enhancing public green infrastructure to mitigate these spatial disparities. Mid-to-high-end commercial residential communities demonstrate deficiencies in facility accessibility and social support systems, necessitating the promotion of home-based care services. Furthermore, compared to their urban-core counterparts, these communities exhibit lower adoption rates of smart technologies (66). Strategic integration of digital solutions to develop technology-mediated social networks is recommended to enhance older adults residents’ social connectivity.

### 5.2 Building AFC in UFA from a resilience perspective

The findings of this study demonstrate that facilities and services significantly and positively influence age-friendliness in UFC. This underscores that enhancing community infrastructure and services is crucial in developing age-friendly environments, effectively meeting the needs of older adults and improving their overall wellbeing. This result aligns with existing research, emphasizing the role of community facilities and services in promoting physical and mental health of older adults (67, 68). In UFC, where urbanization is still in progress, this effect is particularly pronounced. The more adequate infrastructure in these areas, compared to rural regions, is an important factor in attracting older adults to reside there (69). At the same time, existing research indicates that basic infrastructure such as benches is an important factor influencing the frequency of social interactions among the older adults (70). Therefore, UFC should improve the construction of barrier-free facilities, create a safe community environment suitable for older adults residents, and add infrastructure such as elevators, street lighting, resting benches, and anti-slip roads to ensure the safety of the older adults and promote their social interactions, thereby enhancing the stability capacity and age-friendliness of the community (71). Additionally, we found that older adults in geographically remote UFC face difficulties accessing public services such as medical care. Collaborating with healthcare institutions can help improve community resilience of medical services, ensuring better access to medical care for seniors in these areas (72). Further enhancement of community age-friendliness requires strengthening cooperation within the public sector (73).

**Figure 8:**
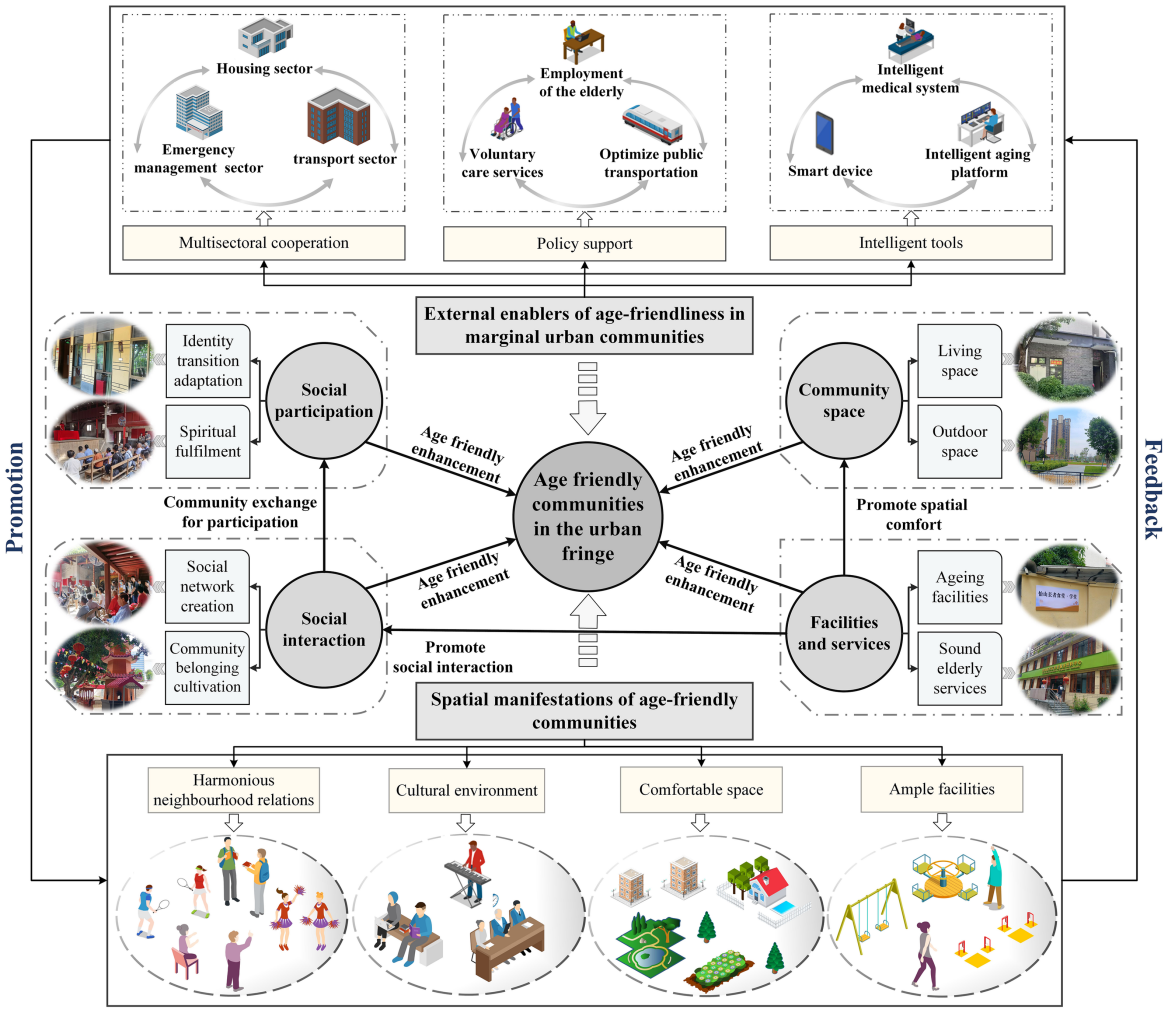
Building AFC in UFA.

Social interaction is another significant factor influencing age-friendliness in UFC. To deepen the positive effects in this area, communities should implement a diverse and high-quality activity system to stimulate deep communication and emotional resonance among older adults (74). Moreover, well-designed small recreational spaces serve as both physical gathering places and key environments for fostering emotional connection and social bonding among the older adults (83). Existing research further highlights how smart technologies can foster social connectivity among older adults, thereby improving their adaptability (75). Therefore, to further promote daily interactions and deeper connections among senior residents in UFC, it is essential for communities to integrate smart elements that bridge the participation gap for older adults in the digital age (76). By helping older adults overcome digital barriers, they can experience the convenience and entertainment brought by smart devices while building new social networks within the community and effectively maintaining existing social systems, thus avoiding emotional estrangement and disconnection (77). The widespread use and application of smart devices serve as a window for older adults to engage with societal trends, allowing them to resonate with mainstream social developments (78). This process enriches the spiritual wellbeing of the older adults and fosters intergenerational communication within families. Shared learning and exploration gradually diminish the “generation gap” that may be exacerbated by technological advancements (79). In addition, learning to use smart devices can play a positive role in responding to global public health events such as the COVID-19 pandemic (80). It can help older adults residents, especially those who have migrated from rural areas, effectively address the challenges of social isolation and difficulties in obtaining supplies caused by the digital divide. Community-based ageing-friendly renovations (such as emergency call devices) and the establishment of mutual aid organizations for the older adults are equally important in responding to public health events. These measures can directly mitigate accident risks among older adults residents, effectively alleviate psychological distress in older adults, and enhance the community’s capacity to maintain stability (81). Figure 8 shows the details of building an AFC in UFA, emphasizing the improvement of both the physical and social environments within communities, as well as external efforts to enhance the age-friendliness of these communities.

## 6 Conclusion

This study defines the concept of AFC in UFA from a resilience perspective, taking into account their specific characteristics. It argues that age-friendliness can be reflected in four dimensions: community space, facilities and services, social interactions, and social participation. This study analyzes the current state of age-friendliness in the UFC of Shangjie Township and develops a structural equation model to identify and quantify the influencing factors. Finally, it proposes a mechanism for the operation of AFC in UFA.

The overall age-friendliness of UFC in Shangjie Township is relatively high, and the level of age-friendliness varies across community types. Community space, facilities and services, social interactions, and social participation all have a positive direct impact on age-friendliness in UFC. Among these factors, the path coefficient for the impact of community facilities and services on age-friendliness is the largest, followed by social interactions, then social participation, with community space having the smallest impact. The operational mechanism of AFC in UFA includes efforts in four aspects: community space, facilities and services, social interactions, and social participation. In contrast to the relatively well-developed infrastructure in urban communities, urban fringe communities suffer from inadequate infrastructure and uneven resource distribution compared to their urban counterparts. Besides, the insufficient resilience of urban fringe communities, compared to their urban counterparts, makes it difficult for older adults residents to adapt and renders them more susceptible to feelings of unease and anxiety. To address these challenges and enhance the age-friendliness of UFC, the government should prioritize fairness in resource allocation at the policy-making level, breaking through the structural constraints of inadequate infrastructure and unequal resource distribution in these communities. Additionally, the government should proactively implement community ageing-friendly renovation policies, mobilize social forces to participate in the construction of community older adults canteens and other infrastructure, and actively respond to local trends such as “re-employment for silver-haired elders” and the “silver economy,” exploring new pathways to support older adults re-employment and ensuring their basic living needs. Furthermore, in this context, further research on the construction of AFC in UFA should focus on establishing a community resilience assessment system tailored to the unique characteristics of such communities, conducting empirical analyses of their association with self-reported health among the older adults, and exploring feasible solutions for smart older adults care platforms in low-resource environments to address the impact of the digital divide on service accessibility.

In sum, this study holds significant theoretical and practical implications. Enhancing the age-friendliness of UFC contributes to improving the quality of life for older adults residents while simultaneously alleviating public health pressures associated with population ageing. However, there are still some limitations about this study. First, there is a general lack of older adults education facilities and day-care institutions in UFC. Although the current survey subjects have not shown a significant demand for or focus on these services, with the deepening wave of urbanization, their potential negative impact on age-friendliness in the future should receive more attention. Furthermore, systematic design of intergenerational shared activity spaces has not yet been incorporated into the assessment system. With the trend toward smaller family structures, its importance will become increasingly apparent. Additionally, considering the heterogeneous characteristics of urban fringe areas how to further acquire and integrate multi-source data for flexible applications in solving complex social problems still requires in-depth exploration in the future.

## Data Availability

The raw data supporting the conclusions of this article will be made available by the authors without undue reservation.

## Footnotes

^1^ https://lbs.amap.com/

## Appendix

### Structural equation model

#### Model formula

Structural equation models consist of measurement models and structural models, which can be represented by three matrix equations. The specific expressions are as follows:

(1)
E[η]=Bη+Γξ+ζ

(2)
$$y=\lambda_{y}\eta +\varepsilon$$

(3)
$$x=\lambda_{x}\xi +\delta$$

Equation 1 is structural model between latent variables. E[·] is the expected value of the latent variable, *η* is the endogenous latent variable, *ξ* is the exogenous latent variable, *B* is the correlation coefficient matrix between endogenous latent variables, *Γ* is the coefficient matrix of the influence of exogenous latent variables on endogenous latent variables, and *ζ* is the measurement error, with *E*[*ζ*] = 0. The endogenous latent variable (*η*) in this study is age-friendliness, while the exogenous latent variables (*ξ*) are community space, facilities and services, social interaction, and social participation.

Equations 2, 3 are measurement models, which are equations used to measure the relationship between latent variables and observed variables, including the measurement of exogenous latent variables and endogenous latent variables. *y* denotes the vector combination of endogenous observed variables, $\lambda_{y}$ is the loading matrix of endogenous latent variables; *x* denotes the combination of exogenous observed variables, $\lambda_{x}$ denotes the loading matrix of exogenous latent variables; *δ* and *ε* are the measurement errors of exogenous and endogenous observed variables, respectively. The endogenous observed variables in this study include community safety, etc., while the exogenous observed variables consist of recovery of space occupancy, completeness of facility types, convenience of information access, and degree of participation in community affairs, among others.

#### Analysis of model results

The reliability and validity tests for the selected variables of community space, facilities and services, social interaction, and social participation yielded the following results: the Cronbach’s alpha coefficient was 0.774 (greater than 0.7), the KMO index was 0.883 (greater than 0.7), and the Bartlett’s approximate chi-square was 1,542.105 with a significance level of *p* < 0.001. The results indicate that the data possesses scientific rigor and consistency. Based on the validity and reliability tests, confirmatory factor analysis was conducted, and the model fit results showed that the chi-square value was less than 3, with the root mean square error of approximation (RMSEA) being less than 0.1, indicating a good overall fit of the model. The incremental fit index (IFI) and comparative fit index (CFI) were both greater than 0.9, reflecting excellent fit, while the normed fit index (NFI) and Tucker-Lewis index (TLI) were slightly below 0.9, suggesting reasonable fit. Therefore, the structural equation model constructed in this study is acceptable and effectively reflects the relationships among the variables.
